# The immunomodulation role of Th17 and Treg in renal transplantation

**DOI:** 10.3389/fimmu.2023.1113560

**Published:** 2023-02-01

**Authors:** Dan-Lei Huang, Yi-Ran He, Yu-Jing Liu, Hong-Yu He, Zhun-Yong Gu, Yi-Mei Liu, Wen-Jun Liu, Zhe Luo, Min-Jie Ju

**Affiliations:** ^1^ Department of Critical Care Medicine, Zhongshan Hospital, Fudan University, Shanghai, China; ^2^ Department of Nursing, Zhongshan Hospital, Fudan University, Shanghai, China; ^3^ Department of Urinary Surgery, Zhongshan Hospital, Fudan University, Shanghai, China

**Keywords:** renal transplantation, T cell, Th17, Treg, Th17/Treg

## Abstract

Kidney transplantation (KT) is an ultimate treatment of end-stage chronic kidney disease, which can meet a lot of complications induced by immune system. With under-controlled immunosuppression, the patient will obtain a good prognosis. Otherwise, allograft disfunction will cause severe organ failure and even immune collapse. Acute or chronic allograft dysfunction after KT is related to Th17, Treg, and Th17/Treg to a certain extent. Elevated Th17 levels may lead to acute rejection or chronic allograft dysfunction. Treg mainly plays a protective role on allografts by regulating immune response. The imbalance of the two may further aggravate the balance of immune response and damage the allograft. Controlling Th17 level, improving Treg function and level, and adjusting Th17/Treg ratio may have positive effects on longer allograft survival and better prognosis of receptors.

## Introduction

Kidney transplantation (KT), as the ultimate treatment of end-stage chronic kidney disease (CKD) (1), same as other solid organ transplantation, can meet a lot of complications induced by immune system. Once immunosuppression is under control, with allograft functioning well, the patient will achieve a relatively high quality of life. Otherwise, allograft disfunction will cause severe organ failure and even immune collapse.

The most valuable evaluation index after renal transplantation is renal function. Routine assessment of graft function usually includes monitoring of serum creatinine levels and screening for proteinuria. Sometimes, allograft biopsy may be required to clarify the abnormality of kidney function. Various immune mechanisms may cause abnormal renal function after renal transplantation. Acute or chronic rejection of allograft may be mediated by T cells, and T-cell–mediated rejection (TCMR) remains a major obstacle to the long-term survival of kidney transplant patients (1–3). It is reported that Th1/Th2 balance is thought to be the main mechanism of rejection (4) However, certain immune events occurring after KT cannot be explained by Th1/Th2 balance alone.

Multiple functions of T cells had been approximately classified into coordinators [i.e., T helper (Th) cells and regulatory T cells (Tregs)] and effectors (i.e., cytotoxic T cells) (5, 6). Th17 cells were first reported in the mechanism of autoimmune diseases, as the additional subsets of Th1 and Th2, and its related cytokines also play an important role in the occurrence of acute and chronic allograft injury after organ transplantation (7, 8). Treg has been confirmed to play a role in regulating tolerance and rejection in animal models of solid organ transplantation (9). Signaling cells can induce T cell differentiate from naïve T cell by secreting kinds of cytokines, a correlation of different subtypes can also affect the procedure, and multiple discovered or undiscovered mechanisms help to maintaining the balance of T-cell–associated immunity. However, changes in the proportions of T-cell subtypes can be observed in renal allograft rejection (4, 10).

In this review, we will briefly describe the differentiation of Th17 and Treg and narrate the relevance between Th17 and Treg. Last, we will discuss the relationship between renal allograft rejection and Th17, Treg, Th17/Treg imbalance, and some possible immunosuppression treatment aimed at them.

## Differentiation of Th17 and Treg

### Th17

Th17 cells are T helper cells that express retinoic acid receptor-related orphan receptor γt (RORγt) and secrete interleukin-17A (IL-17A) and IL-17F cytokine. In the peripheral blood, Th17 was discovered and owned its name because of IL-17, which is the characteristic cytokine of it (11). IL-17 induces a powerful proinflammatory response by stimulating secretion of proinflammatory molecules by combining ubiquitous IL-17 receptor on epithelial cells, endothelial cells, monocytes, and macrophages (12).

IL-6 and transforming growth factor–β (TGF-β) are the critical cytokines for Th17 differentiation, and there are three possible stages of Th17 differentiation in mice: first, combined effect of TGF-β and IL-6/IL-21 triggers differentiation of Th17 cells; then, IL-21 secreted by Th17 cells and TGF-β induced amplification of Th17 cell themselves; and, finally, IL-23 stabilizes Th17 cells (13). Combination of TGF-β and IL-21 has been shown to be sufficient to induce the differentiation of human Th17 cells from immature T cells; meanwhile, IL-1β and IL-6 are important for enhancing the differentiation and memory expansion of Th17 cells (14). Tumor necrosis factor-α (TNF-α) plays an accessory role in Th17 differentiation (15). Signal transducer and activator of transcription 3 (STAT3) plays a key role in positive regulation the differentiation of Th17. After being activated by cytokines such as IL-6, IL-21, IL-23, TNF-α, and TGF-β, STAT3 can upregulate RORγt and promote the differentiation of Th17 (16).

Four distinct mechanisms are described in inhibiting Th17 differentiation: IL-13 can decrease the production of IL-17 by stimulating Th17 cells to produce IL-10, which results in the downregulation of IL-6 (17); IL-27 and IFN-γ through STAT1 activation (12, 18); IL-2 and IL-4 through STAT5 activation (19); and the inhibition of RORγt by Foxp3 (20). Few STAT family members are involved in regulation of Th17 differentiation mediated through some cytokines (16, 18). IL-27, IL-13, and IFN-γ are responsible for inhibiting Th17 development in a STAT1-dependent manner (16, 18, 21, 22). IL-2 also participates in negative regulation of Th17 differentiation through STAT5 (19).

Th17 cells are involved in a variety of autoimmune diseases, including psoriasis, rheumatoid arthritis, inflammatory bowel disease, and multiple sclerosis (23, 24). Meanwhile, Th17 cells can also defend extracellular pathogens, including fungi and bacteria, colonizing the mucosal surface (25). It has been reported that Th17 deficiency can be associated with fungi co-infection, immunoparalysis development, and increased mortality (26–28).

### Tregs

Tregs, either originating from the thymus [natural (n)Treg] or induced peripherally by antigen exposure and cytokines [induced (i)Treg], are CD25+ CD4+ Foxp3+ T cells, continuously expressing cytotoxic T-lymphocyte–associated protein-4 or CD152 and glucocorticoid-inducible tumor necrosis factor receptor (29, 30). Tregs characteristically express Foxp3 and are major immunoregulatory cells with an ability to suppress exaggerated pro-inflammatory action of effector T cells (i.e., activated Th1, Th2, Th3, Th9, Th17, and cytotoxic T cells) (31).

Tregs function by producing the inhibitory cytokines IL-10 and TGF-β (32, 33), interfering with T-cell survival through IL-2 depletion (34), and secreting molecules that directly eliminate effector cells and inhibit antigen-presenting cell maturation and functionality (34, 35). It means that Tregs may show an antagonistic effect against Th17 in an immune response dysregulation individual. TGF-β also plays an important role in the differentiation of Tregs through the induction of STAT5 transcription factor (36). Then, IL-2, through the induction of transcription factor STAT5, and retinoic acid further enhanced the differentiation toward Treg subset (37). In turn, STAT5 will enhance Foxp3 expression. Whereas, retinoic acid can promote TGF-β signaling and Foxp3 promoter activity and can inhibit Th17 differentiation by blocking IL-6 signaling simultaneously (38). IL-10 also plays a part in promoting differentiation of Tregs (39).

It is worth mentioning that, although knockdown of Foxp3 can significantly inhibit Treg function, because Foxp3 is induced upon TCR stimulation, it is possible that Foxp3 expression is not an ideal marker for human Tregs (40). Several lines of evidence suggest that the combination of CD4 and CD25 and the low expression of CD127 identify a subset of peripheral blood T cells, which are highly suppressive in functional assays and are the highest expression of FoxP3, suggesting that the IL-7 receptor (CD127) may be a better biomarker for human Treg (41).

### Th17/Treg

**Figure 1:**
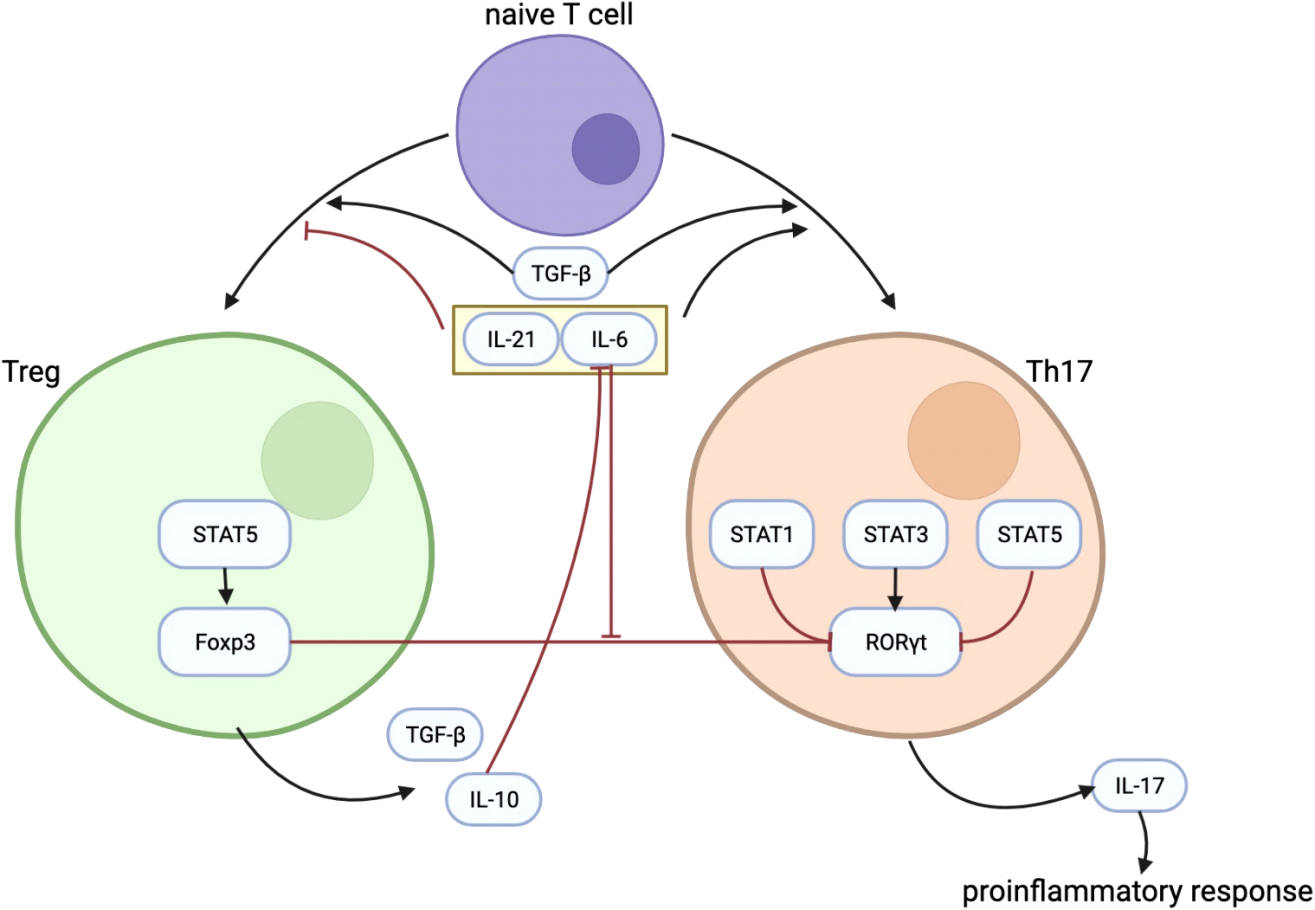
The figure shows some important mechanisms in the differentiation process of Treg and Th17, as well as the interaction between them. TGF-β plays an important role in the differentiation of Treg and Th17. IL-21 and IL-6 could inhibit the differentiation of Treg while promoting the differentiation of Th17. IL-10 secreted by Treg can inhibit the effect of IL-6. Treg regulates Th17 differentiation through the inhibitory effect of Foxp3 on RORγt, and the inhibitory effect of Foxp3 on RORγt is inhibited by IL-6. Treg, regulatory T cells; Th17, T helper cells 17; TGF-β, transforming growth factor–β; IL-21, interleukin-21; IL-6, interleukin-6; IL-10, interleukin-10; Foxp3, forkhead box protein 3; RORγt, receptor-related orphan receptor γt.

Because it has been described that Foxp3 can inhibit RORγt function that turns out to reduce Th17 cell differentiation (20), substantiating the balance between Foxp3 and RORγt is therefore a very important factor in the Th17/Treg balance. Although TGF-β can induce the development of both Tregs and Th17 cell from naïve T cells, Foxp3, induced by TGF-β as well, inhibits Th17 cell differentiation by inhibiting RORγt function when other inflammatory factors are absent (20). TGF-β–induced Foxp3 expression is inhibited by IL-6 (42), IL-21 (43), and IL-23 (20). IL-6 acts as proinflammatory cytokine in T cells by promoting Th17 differentiation and inhibiting Treg differentiation to regulate the balance between Th17 and Treg (18). **Figure 1** shows some important mechanisms in the differentiation process of Treg and Th17, as well as the interaction between Th17 and Treg.

Because Th17 and Tregs play the opposite roles in the immune response and maintain a medium stage of immune activation, which is neither hyperactivation of immune response nor immunosuppression, Th17/Treg imbalance may produce a marked effect in immune dysfunction.

## Relationship between Th17, Treg, and KT

### Th17 and KT

In allograft rejection and dysfunction, it is important to identify the main causes of graft rejection due to the complexity and diversity of mechanisms. Th17 is now known to play a role in both acute allograft rejection and chronic allograft dysfunction.

Some studies have suggested that causes such as ischemia/reperfusion that occurs during transplantation, as well as collagen exposure (Col V), may promote the differentiation of naïve T cells into Th17 under conditions of low levels of TGF-β1 and high levels of IL-6. In addition, Col V is more expressed in bronchial and alveolar tissues. It is assumed that Th17 anti–Col V cell-mediated immunity may be related to graft rejection in lung transplantation (44).

IL-17, an important cytokine secreted by Th17, was found to have increased local expression in graft rejection. In addition, increased infiltration of Th17 cells was significantly associated with incomplete recovery, recurrent TCMR, steroid-resistant rejection, and lower graft survival after rejection (45). Several hypotheses have been proposed. The secretion of IL-17 by Th17 plays a role in the recruitment of neutrophils (46). At the same time, renal epithelial cells exposed to IL-17 produce inflammatory mediators and stimulate the early alloimmune response (47). Th17 cells can also further drive the alloimmune response by promoting lymphoid regeneration (7). Thus, it is assumed that Th17 cells induce a stronger and more durable alloimmune response and result in severe graft tissue damage.

IL-17 induces IL-6, IL-8, monocyte chemoattractant protein-1 (MCP-1), and complement component C3 through the src/mitogen-activated protein kinase pathway (48). In addition, IL-17 exerts its effects through the synergistic interaction with cd40 ligand and the activation of nuclear factor–κB (49).

In the heart transplantation model, antagonism of the IL-17 network (through expression of the IL-17R-immunoglobulin fusion protein) reduced the production of an intra-graft inflammatory cytokine (i.e., IFN-γ) and prolonged graft survival (50).

Studies have found that the Th17 levels in patients who develop CKD after KT are higher than that in patients with normal renal function who undergone KT. In addition, Th17 levels in patients with CKD who have not undergone KT are also lower than those after KT, suggesting that immune response is the cause of the development of CKD after transplantation (45, 51). Retrospective studies have found that the increase in the proportion of Th17 cells is consistent with the increase in the rate of graft failure (52). In addition, Th17 infiltration of allograft has a certain indicator effect on transplantation prognosis and anti-rejection response (53).

### Treg and KT

Treg is considered as an important part in inhibiting activated T-cell function and regulating immunity. The main mechanisms are separated into two types: contact-dependent mechanisms that are dependent on the intercellular receptor and ligand contact, and contact-independent mechanisms that function on the secretion of cytokines (54). It is generally assumed that Tregs act by direct contact with cells, mediated by other active cells or by IFN-γ (55). *In vitro*, Tregs have the ability to inhibit the proliferation and cytokine production of responsive (CD4+ CD25− and CD8+) T cells and downregulate the responses of CD8+ T cells, NK cells, and CD4+ cells to specific antigens (56, 57). *In vivo*, it can play a role in preventing graft rejection (58).

On the basis of the effects of Th1, Th2, and Th17 on the rejection and dysfunction of solid transplanted organs, and the inhibitory effect of Treg on the above cells and their related immune responses, it can be assumed that the increase of Treg level has a certain protective effect on the transplanted organs.

Although the specific mechanism of Treg in promoting human organ transplantation tolerance in terms is unclear, Treg level has an obvious correlation with allograft survival rate (59), and cardiac transplantation related study has found that the local and total Treg and iTreg level is negatively related to the incidence of allograft rejection present (60), prompting that Treg may play a positive role in graft tolerance. In addition, some studies have found that FOXP3 gene hypomethylation may be used as a marker of the percentage of infiltrated Treg in the graft to predict the incidence of rejection events after the suppression of solid organs (61).

### Th17/Treg and KT

As mentioned above, because the changes in the Th17 and Treg levels are related to the occurrence of renal rejection after KT, and on the basis of the interaction between Th17 and Treg, the ratio of local infiltration of Th17/Treg and the balance of Th17/Treg are also theoretically related to transplant organ rejection.

It has been suggested that kidney perfusate–derived extracellular vesicles (KP-EVs) released in allografts may signal the degree of ischemic stress and are considered playing an important role in the development of anti-donor immunity (62, 63). *In vitro* studies confirmed that stimulation of peripheral blood monocytes in this KP-EV environment resulted in a significant reduction in the proportion of Tregs, accompanied by an increase in the Th17/Treg ratio. The expression of miR-218-5p KP-EV increased in allograft of patients with chronic graft rejection. MiR-218-5p KP-EV may participate in the immune process and become a key regulator of T-cell activation through molecular processes, and its expression may be related to the change of Th17/Treg ratio (64).

Some studies have indicated that the imbalance of T-cell subtype proportion is related to the occurrence of CKD in patients after renal transplantation. Compared with normal and mild functional decline individuals, patients with significantly decreased renal function after KT have higher Th17 local infiltration and lower Treg local infiltration of allograft (10). Study has also confirmed that a higher Th17/Treg rate of infiltration in allografts is significantly correlated with decreased allograft function and more grievous interstitial and tubular injury (65).

## Immunosuppression treatment aimed at Th17, Treg, and Th17/Treg

### Th17-related immunosuppression treatment

T cells are inhibited by a combination of tacrolimus (Tac), mycophenolate, and steroids. In addition, induction therapy with the anti-CD25 monoclonal antibody Basiliximab can also inhibit the proliferation of t cells (66). Even if the short-term use of immunosuppressive therapy can avoid most short-term allograft rejection after KT, the long-term prognosis improvement is not ideal (67), suggesting that the current immunosuppressive therapy still has some limitations. Local infiltration of Th17 may lead to chronic allograft dysfunction, and some Th17-inhibiting drugs may be helpful for treatment.

Mammalian target of rapamycin (mTOR) plays an important role in T-cell differentiation, and inhibitors that limit its effect may be beneficial to patients after transplantation. Sirolimus (SRL) has been shown to reduce Th17 levels in patients after renal transplantation. Treatment with SRL instead of Tac can effectively control Th17 levels (68).

1α,25-Dihydroxyvitamin D3 combined with Tac can also play a role in regulating Th17 levels. It has been reported that the combination of the two can significantly inhibit peripheral Th17 and reduce IL-17 and IL-22 levels (69).

### Treg in post-transplantation treatment

The role of Treg in the recovery of patients after KT may mainly lie in several points, promote the recovery of ischemia-reperfusion of transplanted kidney (54), negatively regulate a series of pro-inflammatory factors produced by effector T cells (70), and adjust the level of donor-specific antibodies to regulate humoral immunity (71).

Although part of the immune treatment medicine may play a role in immunosuppression, the limitation is that they may inhibit Treg level (72–75). Therefore, improving the level of Treg in the human body is a kind of auxiliary treatment idea, and achieving this goal means that there are two main methods: (1) Promote Treg proliferation and differentiation endogenously; (2) extract Treg and proliferate *in vitro* and back transfusion.

To promote the proliferation and differentiation of Treg, several drugs have attracted the attention of researchers. In addition to inhibiting Th17 proliferation, mTOR inhibitors can also promote the proliferation and differentiation of Treg (76). The use of alenzumab has also been shown to lead to the production/expansion of Treg (77). Erythropoietin can inhibit the proliferation of other effector T cells while preserving the proliferation of Treg (78). Finally, low-dose recombinant IL-2 is considered as a potential means to enlarge Treg (79).

At present, a series of trials are being conducted for adoptive Treg transplantation in renal transplantation patients. The main technical difficulties lie in how to perform stable and effective amplification after extraction, how to enhance the stability of *in vitro* induced Treg effect, and how to produce specificity for alloantigens during amplification, so as to finally achieve reliable therapeutic effect (54).

### Treatment regulation of Th17/Treg

The changes in Th17 and Treg levels and the imbalance of the two subtypes are related to the allograft dysfunction after KT (10, 65). Adjusting the level of one subtype alone may aggravate the imbalance of the ratio. Therefore, regulating the ratio of the two may also become the research direction of immunosuppression therapy. Currently, there are limited studies on Th17/Treg ratio after renal transplantation as a therapeutic target. However, studies have found that thymoglobulin induction therapy is beneficial to change the ratio of T effector and Treg (80, 81). *In vivo* studies have shown that bortezomib can increase the number of Tregs, can significantly reduce the proportion of Th17 cells, and can also improve renal function and graft survival (82). In rats after KT under carbamylated erythropoietin (CEPO) treatment, it was found that CEPO significantly extended the survival time of the allograft, and flow cytometry showed that Th17/Treg ratio decreased significantly (83). These results indicate that effective treatment can prolong the survival time of kidney grafts, accompanied by the improvement of Th17/Treg ratio.

In recent years, mesenchymal stem cells (MSCs) have attracted more and more attention in the treatment of autoimmune diseases, especially systemic lipus erythematosus (SLE). This therapy can promote the proliferation of Th2 and Tregs; inhibit the activities of Th1, Th17, and B cells; improve the Th17/Treg ratio; and finally improve the signs and symptoms of refractory SLE (84). From the mechanistic point of view, although this kind of cell therapy in patients after transplantation still needs further study support, we can consider MSCs as a potential development direction.

## Conclusions

Acute or chronic allograft dysfunction after KT is related to Th17, Treg, and Th17/Treg to a certain extent. Elevated Th17 levels may lead to acute rejection or chronic allograft dysfunction. Treg mainly plays a protective role on allografts by regulating immune response. The imbalance of the two may further aggravate the balance of immune response and damage the allograft. Controlling Th17 level, improving Treg function and level, and adjusting Th17/Treg ratio may have positive effects on longer allograft survival and better prognosis of receptors.

## Author contributions

Work concept: D-LH, ZL, and M-JJ; Literature collection: D-LH, Y-RH, Y-JL, H-YH, Z-YG, Y-ML, and W-JL; Article writing: D-LH; Mistake correction: D-LH, Y-RH, ZL, and M-JJ; Writing guidance: ZL and M-JJ. All authors contributed to the article and approved the submitted version.
